# Endoscopic salvage of a complex post-endoscopic mucosal resection duodenal perforation

**DOI:** 10.1055/a-2954-0219

**Published:** 2026-09-17

**Authors:** Gregorios A. Paspatis, Magdalini Velegraki, Mathaios Flamourakis, Michael Goumenakis, Nelli Kholcheva, Emmanouil Vardas, Manousos Christodoulakis

**Affiliations:** 1 Department of GastroenterologyCreta InterClinic Hospital-Hellenic Healthcare GroupHeraklionCreteGreece; 2 Department of Medicine 37796 University of IoanninaIoanninaIpiros Ditiki MakedoniaGreece; 3 Department of SurgeryCreta InterClinic Hospital-Hellenic Healthcare GroupHeraklionCreteGreece; 4 Department of RadiologyCreta InterClinic Hospital-Hellenic Healthcare GroupHeraklionCreteGreece

Perforation following duodenal endoscopic mucosal resection (EMR) can be life-threatening and often necessitates surgical intervention. Endoscopic management remains a matter of debate. 1​ 2​ ​3​

We report the case of a 50-year-old woman with a 3-cm 0-IIa adenoma located in the second portion of the duodenum, extending over the ampulla, who was referred for resection. Piecemeal EMR was performed, and the resection margins were extended. The patient was discharged 2 hours after the procedure.

Ten hours later, she was readmitted with severe upper gastrointestinal (GI) bleeding. After resuscitation, upper GI endoscopy demonstrated active bleeding within the post-EMR defect. Hemostasis was attempted using a coagrasper. Careful inspection after hemostasis revealed a limited rupture of the muscularis propria, and endoscopic clips were applied.

Following the procedure, the patient developed abdominal pain. An emergency computed tomographic (CT) scan demonstrated retroperitoneal air. Eighteen hours later, due to persistent pain and recurrent melena, a second emergency upper GI endoscopy was performed. A substantial clot burden was removed from the defect, revealing a large perforation ( Fig. 1 ). Given the friable edges of the defect, an over-the-scope (OTS) clip was deployed as rescue therapy, aiming to grasp the deeper tissue layers rather than the fragile margins ( Fig. 2 ). This approach was selected to avoid immediate surgical repair, which carries considerable morbidity.

**Fig. 1:**
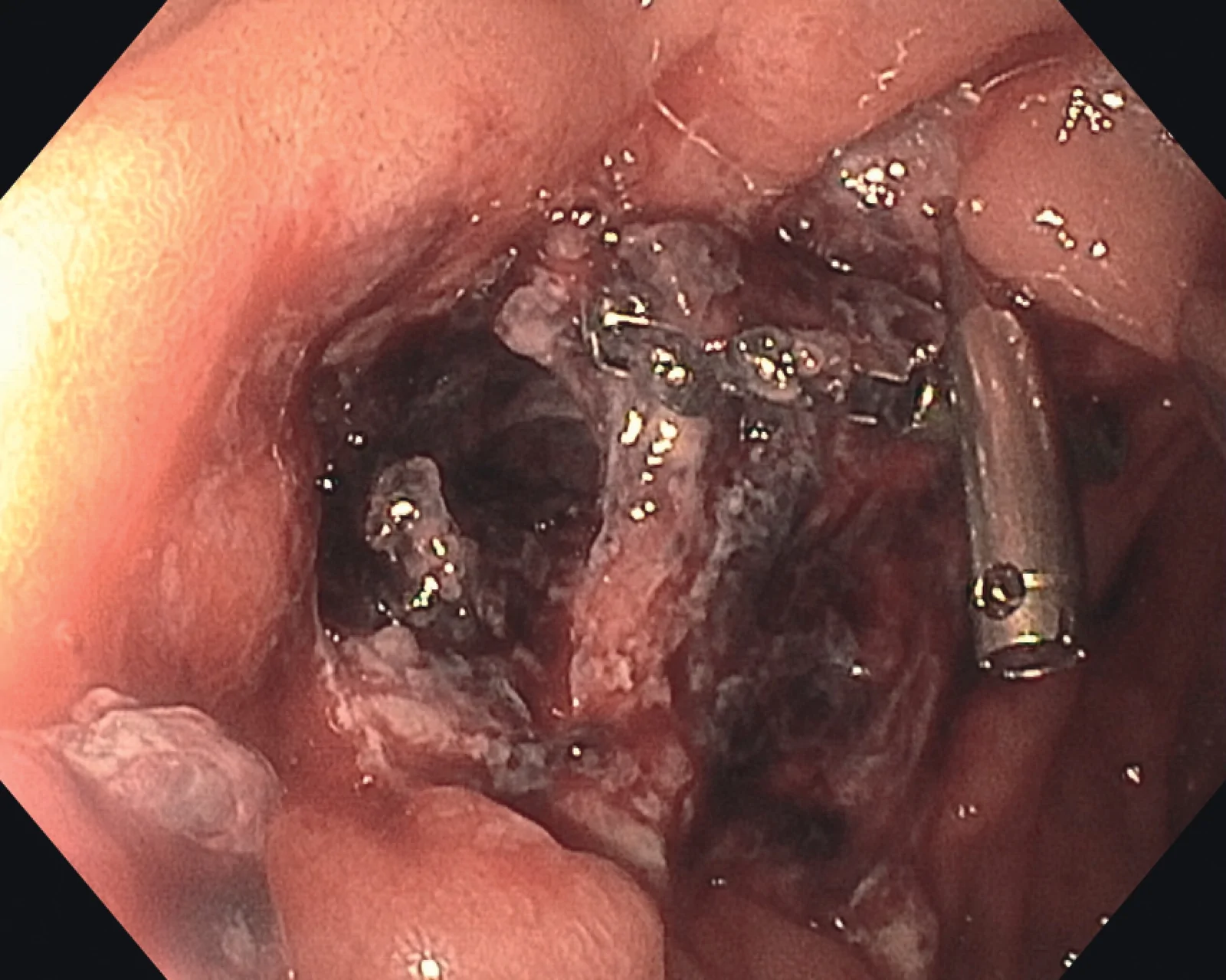
Large duodenal perforation identified within the post-EMR defect before OTS clip deployment.

**Fig. 2:**
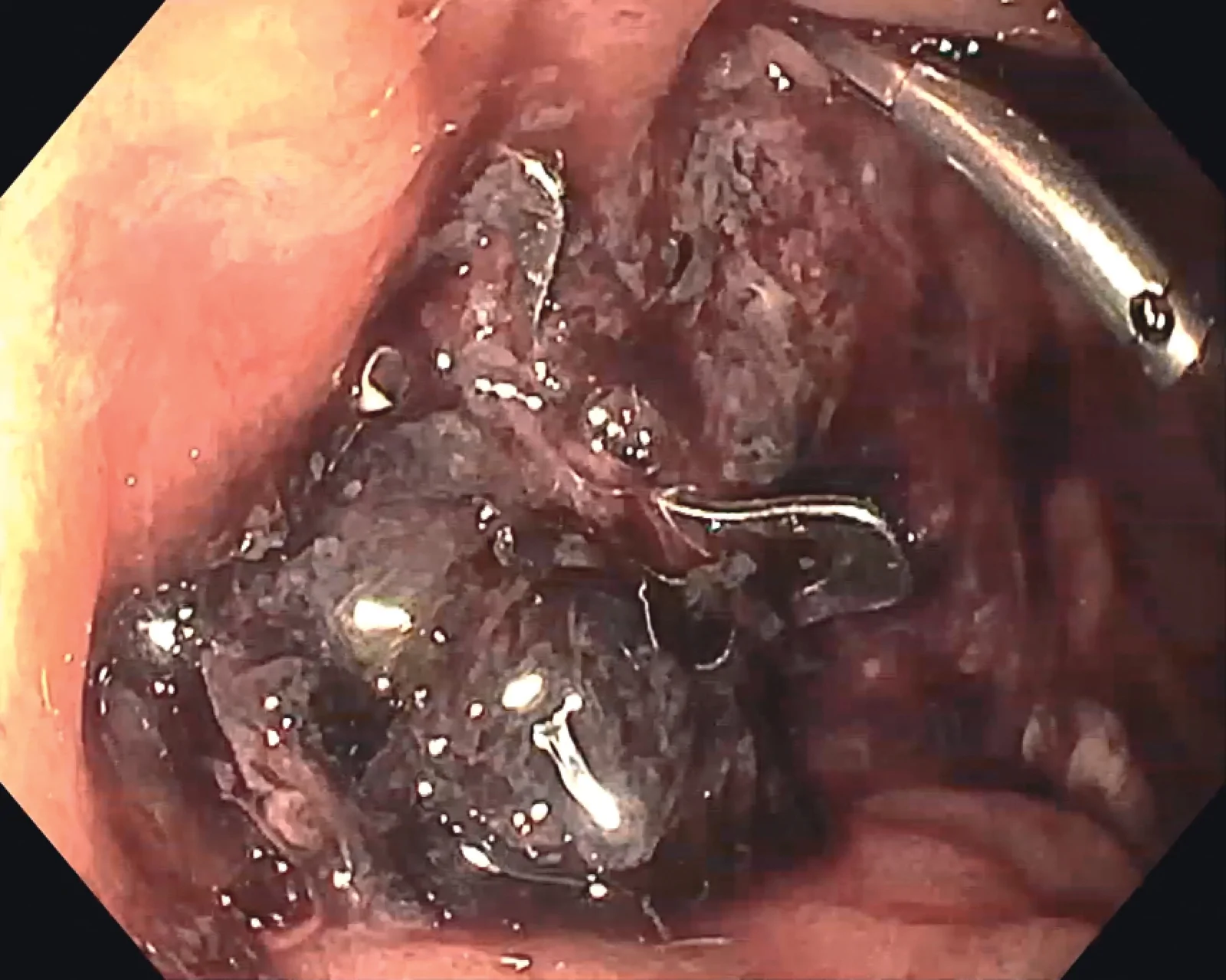
Successful closure of the duodenal perforation after deployment of the OTS clip.

A CT scan with water-soluble contrast performed immediately afterward showed no contrast extravasation at the perforation site, although retroperitoneal gas and fluid persisted. Hemostasis was achieved.

Ten days later, the patient developed fever, and a retroperitoneal fluid collection was successfully drained percutaneously. One month later, she was completely asymptomatic. Six-month follow-up with upper GI endoscopy and CT scan were unremarkable, apart from the retained OTS clip ( Video 1 ).

**Video 1** Endoscopic rescue management of a delayed duodenal perforation after piecemeal EMR of a large adenoma using an OTS clip, achieving successful closure and avoiding surgery. OTS, over-the-scope.


This case highlights the potential of OTS clip deployment as a minimally invasive rescue strategy for complex duodenal perforations after EMR.

Endoscopy_UCTN_Code_CPL_1AH_2AZ_3AC

## Declaration of GenAI Use

The authors declare that no generative artificial intelligence was used in the preparation of this manuscript.

## Data Availability

Data is available within the published article in the Supporting material.
